# National Utilization and Clinical Scenarios of Robotic Appendectomy in the United States, 2022 to 2024

**DOI:** 10.1097/AS9.0000000000000695

**Published:** 2026-08-04

**Authors:** Totadri Dhimal, Anthony Loria, Elen Safarian, Bailey Hilty Chu, Teresa Pullano, Megan Boyer, Michael A. Vella, Fergal Fleming

**Affiliations:** From the * Surgical Health Outcomes Research Enterprise, University of Rochester Medical Center, Rochester, NY; † Department of Surgery, University of Rochester Medical Center, Rochester, NY.

## INTRODUCTION

Appendectomy is among the most commonly performed operations in the United States and is predominantly conducted laparoscopically, the current standard of care.^1^ Despite excellent outcomes associated with laparoscopy, the utilization of robotic appendectomy has increased, prompting questions regarding its clinical value in a high-volume operation.^2^ Existing studies are limited by small sample sizes or insufficient clinical granularity, precluding assessment of whether robotic appendectomy is selectively used in technically challenging or diagnostically complex cases or has diffused into the routine management of uncomplicated appendicitis.^2–4^

We characterized contemporary patterns of minimally invasive (MIS) appendectomy and identified factors associated with the use of a robotic approach. We hypothesized that robotic appendectomy is preferentially used in cases involving greater technical or diagnostic complexity.

## METHODS

We identified adults undergoing MIS appendectomy in the 2022 to 2024 American College of Surgeons National Surgical Quality Improvement Program general and appendectomy procedure–targeted files, corresponding to the introduction of variables specifying a robotic approach.^5^ Procedures were classified as laparoscopic or robotic using operative approach variables, and cases with missing, open, or “other” operative approaches were excluded.

Covariates included demographics, comorbidities, body mass index (BMI), American Society of Anesthesiologists status, case acuity [elective or non-elective (urgent/emergent)], and concurrent procedures. Operative characteristics included unplanned conversion to open or hand-assisted surgery and operative duration.

The primary outcome was operative approach (robotic vs laparoscopic). Secondary outcomes included temporal trends in robotic utilization and 30-day outcomes. Multivariable logistic regression identified factors that are independently associated with a robotic approach, reported as odds ratio (OR) with 95% CI. The severity-adjusted model incorporating appendicitis severity^6^ was restricted to the procedure-targeted file. Missing data were handled using complete-case analysis, and statistical significance was a 2-sided *P* < 0.05.

## RESULTS

Among 148,276 patients undergoing MIS appendectomy, 1.9% (n = 2806) underwent a robotic approach. Robotic utilization increased from 0.9% (n = 486) in 2022 to 3.1% (n = 1512) in 2024. Patients undergoing robotic appendectomy were older, more often female, had higher BMI, were more frequently classified as American Society of Anesthesiologists 3/4, and underwent elective procedures (Table 1).

**TABLE 1.** Baseline Characteristics for Patients Undergoing MIS Appendectomy

Variables, No. (%)	Laparoscopic (n = 145,470)	Robotic (n = 2806)	*P*
Age, y	41.4 (16.8)	46.2 (17.6)	<0.0001
Female	71,670 (49.3%)	1,443 (51.4%)	<0.0001
Race			
Asian	9,349 (6.4%)	132 (4.7%)	<0.0001
Black	9,429 (6.5%)	324 (11.5%)	
Other	9,040 (6.2%)	273 (9.7%)	
White	81,484 (56.0%)	1,859 (66.3%)	
Missing	36,168 (24.9%)	218 (7.8%)	
Ethnicity			
Hispanic	26,598 (18.3%)	609 (21.7%)	<0.0001
Unknown	27,576 (19.0%)	91 (3.2%)	
Body mass index, mean (SD)	25.9 (11.4)	29.3 (9.5)	<0.0001
Diabetes	9,732 (6.7%)	322 (11.5%)	<0.0001
Smoking	16,287 (11.2%)	300 (10.7%)	0.4008
Functional status			
Independent	141,913 (97.6%)	2,732 (97.4%)	0.0027
Partially dependent	543 (0.4%)	22 (0.8%)	
Totally dependent	85 (0.1%)	(0.0%)	
Unknown	2,929 (2.0%)	52 (1.9%)	
COPD	1,343 (0.9%)	48 (1.7%)	<0.0001
Oxygen support	1,509 (1.0%)	26 (0.9%)	0.5659
Heart failure	1,296 (0.9%)	54 (1.9%)	<0.0001
Hypertension	24,894 (17.1%)	779 (27.8%)	<0.0001
Immunosuppressive therapy	2,674 (1.8%)	84 (3.0%)	<0.0001
Dialysis	319 (0.2%)	11 (0.4%)	0.0545
Disseminated cancer	390 (0.3%)	17 (0.6%)	0.0007
Pre-op sepsis			
None	101,353 (69.7%)	2,301 (82.0%)	<0.0001
SIRS	34,002 (23.4%)	407 (14.5%)	
Sepsis	9,655 (6.6%)	94 (3.3%)	
Septic shock	460 (0.3%)	4 (0.1%)	
ASA physical status			
ASA 1-2	114,387 (78.6%)	1,953 (69.6%)	<0.0001
ASA 3-4	30,722 (21.1%)	850 (30.3%)	
Other	361 (0.2%)	3 (0.1%)	

ASA indicates American Society of Anesthesiologists; COPD, chronic obstructive pulmonary disease; SIRS, systemic inflammatory response syndrome.

Within the appendectomy-specific file, 42,232 laparoscopic and 711 robotic cases were identified. Robotic cases had higher rates of complicated appendicitis (27.3% vs 24%) and nonappendicitis pathologies (18.3% vs 7.2%; Supplemental Table 1, see https://links.lww.com/AOSO/A650). On multivariable analysis, factors associated with robotic appendectomy were elective cases [OR, 3.10 (95% CI, 2.61–3.69)], higher BMI [OR, 1.26 per 10-kg/m^2^ increase (95% CI, 1.13–1.38)], malignant pathology [OR, 2.08 (95% CI, 1.49–2.91)], concurrent procedures [OR, 1.49 (95% CI, 1.17–1.89)], and complicated appendicitis [OR, 1.63 (95% CI, 1.34–1.98)]. Postoperative outcomes were similar between approaches, while operative duration was longer with the robotic approach.

## DISCUSSION

Robotic appendectomy remains uncommon but increased between 2022 and 2024. Its use was strongly associated with elective operations, higher BMI, malignant or nonappendicitis pathology, concurrent procedures, and complicated appendicitis. These findings suggest that robotic appendectomy is currently applied selectively in cases with greater technical or diagnostic complexity rather than diffusing into routine management of uncomplicated appendicitis.

The associations with malignant pathology, higher BMI, concurrent procedures, and elective status support preoperative selection based on anticipated operative difficulty, formal resection, or combined operations. Although meta-analyses across oncologic and nononcological procedures suggested lower conversion rates with the robotic platform, we observed no significant differences in conversion to hand-assisted or open surgery or in 30-day postoperative outcomes between approaches.^7,8^ These findings reinforce laparoscopic appendectomy as the standard of care for uncomplicated appendicitis.

This study has limitations. The National Surgical Quality Improvement Program does not capture surgeon identifiers, institutional robotic resources, or detailed cost data, precluding assessment of learning curves, surgeon-level selection, geographic variation, and economic value. Prior studies have shown that robotic appendectomies have a longer operative time and higher costs than laparoscopy.^9^ Given its low utilization, robotic appendectomy has limited population-level resource implications. However, increasing adoption of robotic platforms has intensified competition for finite robotic operating room time. In this context, longer operative duration carries opportunity costs, including reduced operating room efficiency and diminished access for procedures that may derive greater outcome benefit from robotic technology. These considerations are particularly relevant for appendectomy, a high-volume operation with excellent outcomes using conventional laparoscopy.

Together, our findings support the role of robotic surgery as a selective adjunct for appropriately chosen patients rather than a routine replacement for laparoscopic appendectomy. Thoughtful preoperative selection may maximize the value of robotic platforms while preserving access for cases most likely to benefit from their technical advantages.

## Supplementary Material



## References

[1] Podda M Ceresoli M De Simone B. Diagnosis and treatment of acute appendicitis: 2025 edition of the World Society of Emergency Surgery Jerusalem guidelines. JAMA Surg. 2026;161:283–295. 10.1001/jamasurg.2025.6218 41604201

[2] Mullens CL Savitch SL Napolitano LM. Uptake of robotic appendectomy: a single state analysis. Ann Surg. 2026;283:911–913. 10.1097/SLA.0000000000007004 PMC12973531 41423774

[3] Becker T DeLeon G Rao V. A comparison of outcomes between laparoscopic and robotic appendectomy among ACS-NSQIP hospitals. Laparosc Endosc Robot Surg. 2023;6:39–42.

[4] Thomas EM Orcutt G Norris AE. Comparative analysis of laparoscopic and robotic appendectomy: a multi-hospital retrospective cohort study. Surg Endosc. 2026;40:1362–1367. 10.1007/s00464-025-12419-4 41261248

[5] ACS NSQIP participant use data file. American College of Surgeons. Available at: https://www.facs.org/quality-programs/data-and-registries/acs-nsqip/participant-use-data-file/. Accessed February 17, 2026.

[6] Childers CP Selzer DJ Green ML. Generating a New CPT code set for adult and pediatric appendectomy. JAMA Surg. 2025;160:1076–1081. 10.1001/jamasurg.2025.2951 PMC12368789 40833763

[7] Ricciardi R Seshadri-Kreaden U Yankovsky A. The COMPARE Study: comparing perioperative outcomes of oncologic minimally invasive laparoscopic, Da Vinci robotic, and open procedures: a systematic review and meta-analysis of the evidence. Ann Surg. 2025;281:748–763. 10.1097/SLA.0000000000006572 PMC11974634 39435549

[8] Gangemi A Ebadinejad A Lisi AP. The CONVERSION Study: open conversion risk in robotic vs laparoscopic surgery—a 20-year meta-analysis [published online ahead of print November 14, 2025]. *Ann Surg*. doi:10.1097/SLA.0000000000006976. 10.1097/SLA.0000000000006976 41233947

[9] Quilici PJ Wolberg H McConnell N. Operating costs, fiscal impact, value analysis and guidance for the routine use of robotic technology in abdominal surgical procedures. Surg Endosc. 2022;36:1433–1443. 10.1007/s00464-021-08428-8 33835252

