# Supplementary material for: National Utilization and Clinical Scenarios of Robotic Appendectomy in the United States, 2022 to 2024
Source: Ann Surg Open. 2026 Aug 4;7(3):e695. doi: 10.1097/AS9.0000000000000695 (PMC13596949; doi:10.1097/AS9.0000000000000695)
Supplement: Supplementary file 1 [file as9-7-e695-s001.pdf]

Supplemental Table 1: Operative Variables and Outcomes

Variables, No. (%)	Non-elective (Urgent/Emergent) Laparoscopic n=118,623	Non-elective (Urgent/Emergent) Robotic n=1,406	p	Elective Laparoscopic N=26,847	Elective Robotic N=1400	p
Surgery in 2022	39,366 (33.2%)	198 (14.1%)	<.01	9698 (36.1%)	270 (19.3%)	<0.01
Surgery in 2023	39,807 (33.6%)	353 (25.1%)		8857 (33.0%)	473 (33.8%)	
Surgery in 2024	39,450 (33.3%)	855 (60.8%)		8292 (30.9%)	657 (46.9%)	
**Days from admission to operation, mean (SD)**	0.3 (2.1)	0.3 (2.2)	0.39	---	---	---
**Unplanned conversion to open/hand assistance**	3,433 (2.9%)	22 (1.6%)	<0.01	772 (2.9%)	46 (3.2%)	0.37
**Operative time, mean (SD), min**	51.1 (33.4)	66.4 (36.3)	<.01	53.4 (34.7)	78.7 (47.3)	<0.01
**Concurrent procedure**	6603 (5.6%)	82 (5.8%)	0.66	2,187 (8.1%)	199 (14.2%)	<0.01
Cholecystectomy	58 (0.05%)	2 (0.1%)	0.12	89 (0.3%)	11 (0.8%)	0.01
Inguinal Hernia	42 (0.04%)	4(0.2%)	<0.01	21 (0.08%)	12 (0.9%)	<0.01
Ventral Hernia	443 (1.7%)	21 (1.5%)	0.34	1,329 (1.1%)	11 (0.8%)	0.0.68
Enterolysis	1,870 (1.6%)	17 (1.2%)	0.29	528 (2.0%)	50 (3.6%)	<0.01
Colectomy	303 (0.3%)	3 (0.2%)	0.75	155 (0.6%)	17 (1.2%)	0.01
Gynecologic procedure	52 (0.0%)	(0.0%)	0.43	28 (0.1%)	9 (0.6%)	<0.01
**Clavien-Dindo Classification**						
None	113,610 (95.8%)	1,351 (96.1%)	0.72	25,921 (96.6%)	1,362 (97.3%)	0.37
Grade 1-2	3,927 (3.3%)	40 (2.8%)		722 (3.0%)	32 (2.3%)	
Grade 3-4	994 (0.8%)	14 (1.0%)		187 (0.7%)	6 (0.4%)	
Grade 5	92 (0.1%)	1 (0.1%)		17 (0.1%)	0 (0.0%)	
**30-day Readmission**	3,976 (3.4%)	57 (4.1%)	0.15	800 (3.0%)	43 (3.1%)	0.84
**30-day Reoperation**	951 (0.8%)	14 (1.0%)	0.42	205 (0.8%)	11 (0.8%)	0.93
**Length of Stay, mean (SD)**	1.6 (3.9)	2.0 (3.9)	<0.01	1.1 (3.7)	0.9 (1.9)	0.05
**Operative Indication****	**n=35,503**	**n=374**		**n=6,729**	**n=337**	
Uncomplicated Appendicitis	25,390 (71.5%)	266 (71.1%)	0.37	3,939 (58.5%)	133 (39.5%)	<0.01
Complicated Appendicitis	8,627 (24.3%)	97 (25.9%)		1,199 (17.8%)	85 (25.2%)	
Malignancy	346 (1.0%)	5 (1.3%)		493 (7.3%)	47 (13.9%)	

Other pathology	1,026 (2.9%)	6 (1.6%)		1,046 (15.5%)	71 (21.1%)	
Unknown	114 (0.3%)	0 (0.0%)		52 (0.8%)	1 (0.3%)	

*Select few procedures of interest from list of concurrent procedures
** Operative indications derived from appendectomy specific PUF, thus have different sample size.
